# Edge-Computing-Based Intelligent IoT: Architectures, Algorithms and Applications

**DOI:** 10.3390/s22124464

**Published:** 2022-06-13

**Authors:** Xiao Liu, Jiong Jin, Fang Dong

**Affiliations:** 1 School of Information Technology, Deakin University, Geelong, VIC 3220, Australia; 2 School of Science, Computing and Engineering Technologies, Swinburne University of Technology, Melbourne, VIC 3122, Australia; jiongjin@swin.edu.au; 3 Department of Computer Engineering, School of Computer Science and Engineering, Southeast University, Nanjing 211189, China; fdong@seu.edu.cn

With the rapid growth of the Internet of Things (IoT), 5G networks and beyond, the computing paradigm for intelligent IoT systems is shifting from conventional centralized-cloud computing to distributed edge computing. Edge computing (i.e., fog computing) can effectively address the critical challenge of high latency faced by cloud computing, by provisioning computing resources close to the IoT devices where massive data are generated. More importantly, edge computing inherits the benefits of cloud services and emphasizes collaboration among cloud servers, edge servers, and end devices to achieve optimized performance. However, there are still many open issues in edge-computing-based intelligent IoT systems.

We invited authors to submit their latest works on the investigation of fundamental issues in edge-computing-based intelligent IoT systems, from three perspectives: architectures, algorithms, and applications. In total, five papers were accepted for publication in this Special Issue of *Sensors*. These papers can be divided into two main categories: task and resource management for edge computing, and edge-computing-based smart IoT systems.

For the first category—task and resource management for edge computing—Yang et al. [1] investigated the problem of task offloading for mobile edge-computing networks; they proposed a deep-supervised-learning-based computational offloading (DSLO) algorithm to jointly optimise the problems of offloading decisions and bandwidth allocation. Rosenberger et al. [2] studied the problem of resource allocation in the industrial Internet of Things (IIoT); they proposed a multi-agent deep-reinforcement-learning (MARL)-based strategy which can deal with several dynamic changes in the target system and achieve the optimal usage of available resources for IIoT devices.

For the second category in edge-computing-based smart IoT systems, Qayyum et al. [3] proposed a data-collection scheme and scheduling framework for smart farms wherein unmanned aerial vehicles (UAVs) are employed to facilitate data collection due to their remote mobility. Iacobescu et al. [4] investigated the problem of end-user satisfaction in the smart parking system, wherein users are often forced to use multiple interfaces to find a parking spot in a geographical area; they tried to solve the problem by proposing a trustless federated model that will facilitate user adoption and responsible data-acquisition by leveraging a federated identity protocol based on Zero-Knowledge Cryptography. Andreadis et al. [5] proposed and evaluated a framework for automatically detecting illegal tree-cutting activity in forests through audio event classification. Specifically, they envisaged tiny ultra-low-power devices, embedding edge-computing microcontrollers and long-range wireless communication to cover vast areas in the forest; additionally, an efficient and accurate audio-classification solution based on convolutional neural networks was proposed to reduce the energy footprint and resource consumption.

We would like to thank the authors for submitting their excellent works to our Special Issue, and appreciate the reviewers for providing their invaluable comments to improve the quality of these papers. We hope this Special Issue can provide some useful research findings and interesting applications to both researchers and practitioners who are interested in edge-computing-based intelligent IoT systems.

## Data Availability

Not applicable.
